# Reference management software for systematic reviews and meta-analyses: an exploration of usage and usability

**DOI:** 10.1186/1471-2288-13-141

**Published:** 2013-11-15

**Authors:** Diane L Lorenzetti, William A Ghali

**Affiliations:** 1 Department of Community Health Sciences, University of Calgary, 3280 Hospital Drive NW, Calgary T2N 4Z6 Alberta, Canada

**Keywords:** Meta-analysis, Systematic reviews, Data collection, Databases, Bibliographic, Software, Reference management software

## Abstract

**Background:**

Reference management software programs enable researchers to more easily organize and manage large volumes of references typically identified during the production of systematic reviews. The purpose of this study was to determine the extent to which authors are using reference management software to produce systematic reviews; identify which programs are used most frequently and rate their ease of use; and assess the degree to which software usage is documented in published studies.

**Methods:**

We reviewed the full text of systematic reviews published in core clinical journals indexed in ACP Journal Club from 2008 to November 2011 to determine the extent to which reference management software usage is reported in published reviews. We surveyed corresponding authors to verify and supplement information in published reports, and gather frequency and ease-of-use data on individual reference management programs.

**Results:**

Of the 78 researchers who responded to our survey, 79.5% reported that they had used a reference management software package to prepare their review. Of these, 4.8% reported this usage in their published studies. EndNote, Reference Manager, and RefWorks were the programs of choice for more than 98% of authors who used this software. Comments with respect to ease-of-use issues focused on the integration of this software with other programs and computer interfaces, and the sharing of reference databases among researchers.

**Conclusions:**

Despite underreporting of use, reference management software is frequently adopted by authors of systematic reviews. The transparency, reproducibility and quality of systematic reviews may be enhanced through increased reporting of reference management software usage.

## Background

Various software programs have been adopted by, and specifically developed for, authors of systematic reviews. These tools range from statistical software to comprehensive systematic reviews programs, such as the Cochrane Collaboration’s Review Manager (RevMan) software. Reference management software programs, including EndNote, RefWorks and Zotero, are one such tool.

Reference management software, also known as bibliographic software, citation management software, or personal bibliographic file managers is “any software product used for storage and retrieval of bibliographic records” [1]. First developed in the 1980s, these programs were initially marketed to researchers as a means of creating online indexes of personal print-article collections [2-4]. As electronic databases, such as MEDLINE, became generally accessible and more easily searchable, researchers began to use this software to maintain databases of all research relevant to their fields of interest [3].

Numerous reference management programs are currently available. Although all programs facilitate the capture, organization, and elimination of duplicate records from electronic database searching, they vary with respect to cost, overall functionality, and networking capabilities. Products such as EndNote, Papers, and RefWorks are licensed or sold outright, while others (e.g.: Mendeley and Zotero), are available at little or no cost to the user. While some, such as EndNote and Reference Manager, run on single-station computers, many others, including RefWorks, Mendeley and Zotero, are web-based. Single-station software usage is generally not affected by website time-lags, down times, or record limits, all of which may impinge on the usability of web-based products. That said, the benefits of these web-based programs include the ability to store reference databases on secure servers, and access databases from multiple computers or other electronic devices. Web-based programs also provide users with enhanced networking functions that readily support the sharing of records among researchers [3,5].

The identification, collection, and organization of relevant studies are instrumental to the successful completion of systematic reviews. To this end, a “rigorous data management plan” is essential [6]. Many guides to undertaking systematic reviews, including the *Cochrane Handbook for Systematic Reviews of Interventions*, recommend reference management software as a means of assisting in the organization and selection of component studies for inclusion in these reviews [6,7].

In the context of systematic reviews, reference management programs facilitate the capture and organization of studies identified through electronic database searching, the identification and elimination of duplicate records from multiple database searches, the transfer of references to Cochrane RevMan and other systematic reviews software, and the accurate citing of references within manuscripts [8,9]. Thus, an author’s decision to use, or not use, this software may impact on the accurate reporting of the number of studies reviewed for inclusion and exclusion in a systematic review.

The purpose of this study was to determine the extent to which authors are using reference management software to produce systematic reviews; identify which programs are used most frequently and rate their ease of use; and assess the degree to which software usage is documented in published studies.

## Methods

We reviewed the full text of systematic reviews published in core clinical journals. We surveyed the corresponding authors of included studies to verify and supplement information in published reports, and gather frequency and ease-of-use data on specific reference management programs. This study received ethics approval from the University of Calgary’s Conjoint Health Ethics Board.

### Study identification

We retrieved the full-text reports of all systematic reviews indexed in the ACP Journal Club from 2008 to November 2011. ACP Journal Club indexes over 100 core clinical journals. Our decision to study systematic review articles featured in ACP Journal Club relates partially to the clinical importance and visibility of these featured articles. We wanted our study to focus on articles that the scientific community has judged to be important. Systematic reviews indexed in ACP Journal Club must meet standard quality criteria including a clear statement of research, and a description of the methods used to identify and select studies for inclusion in the review. By restricting our data collection to studies indexed in ACP Journal Club, we recognize that we have been sampling reviews with better-than-average reporting.

Studies were included if they were English language systematic reviews or meta-analyses. Studies were excluded if they were: (1) meta-analyses of data not obtained through reviews of the published literature (e.g.: chart reviews); (2) systematic reviews where only 1 electronic database was searched to identify relevant studies; or (3) publications where the email addresses of corresponding authors were not provided, or could not be determined. We searched PubMED, Scopus, Web of Knowledge, Google, and Google Scholar to identify current email addresses for corresponding authors of studies selected for review.

### Data collection

We undertook a content analysis of included reviews to identify those authors who had reported on the use of reference management software in their published reviews. As a counterpoint, we also noted the frequency of reporting of statistical software usage. An Excel form was created to extract the following data from each study: author, date, title, source, corresponding author’s name and email address, and documented use of reference management and statistical software. Where authors were listed as corresponding authors in more than one publication, only the most recent study was selected for review.

A survey was emailed to all corresponding authors to compare actual with reported usage, and gather data on specific software programs. The five-item survey asked authors to: (1) indicate whether or not a reference management program had been used; (2) provide the name of the program; (3) rate its functionality on a scale of 0 to 10, with 0 being the “least functional” and 10 the “most functional”; (4) indicate their intention to use this product in future reviews; and (5) provide any additional comments on the usability of this software. Two reminders were sent to corresponding authors at one-month intervals.

### Data analysis

Descriptive statistics were calculated using SPSS V20 statistical analysis software. Responses to the final open-ended survey question (Q#5) were analyzed to identify common themes.

## Results

From a total of 163 systematic reviews identified in ACP Journal Club, 111 authors, representing 122 papers, were included in this study. Based on eligibility criteria, 41 papers were excluded from this study. Reasons for exclusion were: an inability to determine or verify the email address of the corresponding author (n = 30); study authors only searched 1 electronic database to identify studies to include in their systematic review (n = 8), or no electronic databases were searched (n = 3). Of the 111 surveys that were emailed to authors, 78 surveys were returned, for a response rate of 70.27%.

A total of 79.5% (62/78) of respondents indicated that they had used a reference management software program in preparing their reviews. Of these, 4.8% (3/78) had included this information in their published reviews. In comparison, 76.9% (60/78) of authors reported on the use of statistical software in their published reviews (Table 1).

**Table 1:** Software usage and reporting among 78 respondents

	**Reference management software frequency**	**Reference management software percent**	**Statistical software frequency**	**Statistical software percent**
Usage	62	79.5%	78	100%
Reported	3	4.8%	60	76.9%

The reference management software program used most frequently was EndNote (n = 41), followed by Reference Manager (n = 14), RefWorks (n = 6) and Excel (n = 1). Mean functionality ratings for these tools ranged from 7.0 to 8.0 (Table 2).

**Table 2:** Reference software usage by type and functionality among 78 respondents

	**Frequency**	**Percent**	**Mean functionality rating**
EndNote	41	52.6%	7.5 (range 3 to 10)
Reference Manager	14	17.9%	7.0 (range 6 to 9)
RefWorks	6	7.7%	7.8 (range 6 to 10)
Excel	1	1.3%	8.0
None	16	20.5%	NA
Total	78	100%	

Of the 62 authors who reported using reference management software, 4 (2 EndNote and 2 Reference Manager) stated that they would be switching to a different software package in future reviews. Stated reasons for switching software packages included: incompatibility issues between Reference Manager and Mac computers, difficulty importing references into EndNote, and, in one instance, a cost savings that would result from replacing EndNote with Zotero.

Forty-seven authors (60.3%) responded to our final open-ended survey question asking for comments on the usability of reference management programs. Thirteen authors remarked on the ease of use and overall importance of their chosen tool. As one respondent stated:

“I don’t think you can write manuscripts for prestigious journals without one of these bibliography management programs…..there will always be recommended revisions, or a rejection… in which case you’ll have to reconfigure your references. To do [this] by hand is too cumbersome and time-consuming”.

A number of authors also identified specific challenges associated with these programs. Among these were: record errors that occurred when downloading references from electronic databases, such as MEDLINE (n = 4), difficulties in identifying and deleting duplicate records from reference management databases (n = 2); PC/Mac-incompatibilities for users of EndNote and Reference Manager (n = 3); errors in journal output styles (n = 3); difficulties in transferring reference databases from one software package to another (n = 2), and delays in accessing RefWorks databases (n = 2). Five respondents indicated that they were not only using this software to collate studies identified through database searching, but also to record reviewers’ decisions with respect to the inclusion or exclusion of studies.

## Discussion

Our study reveals that most (but not all) authors of systematic reviews are using reference management software tools. Despite this, only a small minority are reporting this usage in their published studies. Further, there appears to be no apparent relationship between choice of reference management software and perceived functionality or usability. Nor was software choice associated with the degree to which usage was reported or not reported in published studies.

Currently, there are more than 28,000 scholarly journals in active publication [10]. Collectively, these journals publish approximately 1.8 million articles per year [10]. Given the trend, across disciplines, towards integrating evidence into daily practice, it is unsurprising that an increasing number of systematic reviews are being funded and produced. Previous research has demonstrated that authors typically search multiple electronic databases to identify studies relevant for systematic reviews [11-14]. Estimates of overlap between these databases have ranged between 8% and 60% [11-14]. Software programs that can facilitate the collection, organization and removal of duplicate studies from database searches are invaluable in the production of large-scale systematic reviews.

The most significant finding from this study was the disparity between stated and reported software use. Few (4.8%) authors who had used reference management software reported this information in their published manuscripts. As a point of contrast, 76.9% of the authors in our sample reported on the statistical software packages used to analyze study results. A recent study on the use of statistical software in health services research also found that a significant number of authors were including statistical software information in their published manuscripts [15]. This discrepancy in reporting may be the result of an absence of guidelines which specifically address this aspect of reporting. Whereas The International Committee of Medical Journal Editors’ *Uniform Requirements for Manuscripts Submitted to Biomedical Journals* recommend that authors “specify the computer software” when describing the statistical methods they employed to analyze study data, no mention is made, here or elsewhere, of the importance of reporting on the use of reference management software [16].

Although the PRISMA checklist for the quality reporting of systematic reviews does not specifically suggest that authors report on reference management software usage, its use can impact the number of unique studies identified and reported in a systematic review [17]. A failure to identify duplicate records captured in database searches may result in an over-reporting of irrelevant studies. Similarly, an over-reliance on reference management software to identify and remove duplicate records from a reference database may cause relevant studies to be overlooked. As such, reference management software can have a real impact on the quality of a completed review.

Whereas numerous studies have been published citing the benefits of reference management and other sofware programs in the production of systematic reviews, the degree to which these programs have been adopted by the research community is largely unknown [2,3,6,9,18-22]. In 2007, Senarath published a research letter on the reference management software experiences of 22 researchers in Sri Lanka [8]. Although informative in terms of highlighting the functions and perceived advantages of this software, to our knowledge, ours is the first study to explore reference management software usage and reporting among authors of systematic reviews.

Our study has caveats and limitations. Although we were able to achieve a strong response rate of 70% from our email survey, a telephone survey could have yielded a higher response rate. Furthermore, researchers who declined to participate in our study may have different perspectives on reference management software usage than those who participated. Secondly, as mentioned previously, we limited our study sample to clinical reviews published in ACP Journal Club. This sampling frame may have resulted in the identification of published systematic reviews that have a better-than-average quality of reporting. Thirdly, an analysis of systematic reviews produced in languages other than English, and other disciplines, such as education or social work, could yield different findings. Finally, with the exception of one open-ended question, our survey did not seek to identify perceived benefits and deficits with respect to individual software programs.

An overriding caveat to our study, and its implications in terms of the importance of reporting reference management software usage, is that there is, as yet, no firm evidence to suggest that reference management software can have a positive impact on the quality of completed reviews. That said, the “value of a systematic review depends on what was done, what was found, and the clarity of reporting” [17]. As stated by Moher and colleagues “the reporting and conduct of systematic reviews are, by nature, closely intertwined” [17]. As such, a detailed transparent plan for the identification and selection of component studies, including the use or lack of use of reference management software, is an important element of any review protocol. It can reflect the methodological expertise of the authors, and the transparency, reproducibility and, ultimately, the quality of the study that has been produced.

## Conclusions

Despite being underreported, reference management software is frequently used by authors of systematic reviews. The transparency, reproducibility and quality of systematic reviews may be enhanced through increased reporting of reference management software usage.

## Competing interests

The authors declare that they have no competing interests.

## Authors’ contributions

DLL contributed to the study design, collected and analyzed the data, and prepared and approved the final version of this paper. WAG conceived of the study, contributed to the study design, and prepared and approved the final version of this paper. Both authors read and approved the final manuscript.

## Pre-publication history

The pre-publication history for this paper can be accessed here:

http://www.biomedcentral.com/1471-2288/13/141/prepub

## References

[B1] Nashelsky J Earley J Reference management software: selection and uses Libr Software Rev 1991 10 174 178

[B2] Garfield JM Flanagan H Fox J A comparison of two microcomputer-based programs for bibliographic retrieval and formatting J Clin Monit 1989 5 177 185 10.1007/BF01627450 2769316

[B3] Gilmour R Cobus-Kuo L Reference management software: a comparative analysis of four products Issues Sci Technol Libr 2011 66 doi:10.5062/F4Z60KZF

[B4] Wachtel R Personal bibliographic databases Science 1987 235 1093 1096 10.1126/science.235.4792.1093 17782260

[B5] Bibliographic management 2.0 [http://www.ukeig.org.uk/elucidate/issue/feature-article-bibliographic-management-20]

[B6] King R Hooper B Wood W Using bibliographic software to appraise and code data in educational systematic review research Med Teach 2011 33 719 723 10.3109/0142159X.2011.558138 21854149

[B7] Cochrane Handbook for Systematic Reviews of Interventions Version 5.1.0 [updated March 2011] [http://www.cochrane-handbook.org]

[B8] Senarath U Bibliographic referencing made easy: use of bibliographic software in health research Ceylon Med J 2007 52 38 39 17585585 10.4038/cmj.v52i1.1053

[B9] Steele SE Bibliographic citation management software as a tool for building knowledge J Wound Ostomy Continence Nurs 2008 35 463 466 10.1097/01.WON.0000335956.45311.69 18794696

[B10] Ware M Mabe M The STM report: an overview of scientific and scholarly journal publishing 2012 The Netherlands

[B11] Betran AP Say L Gulmezoglu AM Allen T Hampson L Effectiveness of different databases in identifying studies for systematic reviews: experience from the WHO systematic review of maternal morbidity and mortality BMC Med Res Methodol 2005 5 6 10.1186/1471-2288-5-6 15679886 PMC548692

[B12] Lohonen J Isohanni M Nieminen P Miettunen J Coverage of the bibliographic databases in mental health research Nord J Psychiatry 2010 64 181 188 10.3109/08039480903337378 19883199

[B13] Royle P Waugh N Literature searching for clinical and cost-effectiveness studies used in health technology assessment reports carried out for the national institute for clinical excellence appraisal system Health Technol Assess 2003 7 iii 64 10.3310/hta7340 14609481

[B14] Royle P Milne R Literature searching for randomized controlled trials used in cochrane reviews: rapid versus exhaustive searches Int J Technol Assess Health Care 2003 19 591 603 15095765 10.1017/s0266462303000552

[B15] Dembe AE Partridge JS Geist LC Statistical software applications used in health services research: analysis of published studies in the U.S BMC Health Serv Res 2011 11 252 10.1186/1472-6963-11-252 21977990 PMC3205033

[B16] Uniform requirements for manuscripts submitted to biomedical journals: writing and editing for biomedical publications[http://www.icmje.org/] 14725274

[B17] Moher D Liberati A Tetzlaff J Altman DG Preferred reporting items for systematic reviews and meta-analyses: the PRISMA statement PLoS Med 2009 6 e1000097 10.1371/journal.pmed.1000097 19621072 PMC2707599

[B18] Bax L Yu LM Ikeda N Moons KG A systematic comparison of software dedicated to meta-analysis of causal studies BMC Med Res Methodol 2007 7 40 10.1186/1471-2288-7-40 17845719 PMC2048970

[B19] Brantz MH Galla J Is there an optimal bibliographic software product for end users? Bull Med Libr Assoc 1988 76 216 220 3416095 PMC227110

[B20] Hernandez DA El-Masri MM Hernandez CA Choosing and using citation and bibliographic database software (BDS) Diabetes Educ 2008 34 457 474 10.1177/0145721708317875 18535319

[B21] Ovadia S Managing citations with cost-free tools Behav Soc Sci Librar 2011 30 107 111 10.1080/01639269.2011.565408

[B22] Wallace BC Schmid CH Lau J Trikalinos TA Meta-analyst: software for meta-analysis of binary, continuous and diagnostic data BMC Med Res Methodol 2009 9 80 10.1186/1471-2288-9-80 19961608 PMC2795760

